# Cell Populations in Human Breast Cancers are Molecularly and Biologically Distinct with Age

**DOI:** 10.21203/rs.3.rs-5167339/v1

**Published:** 2024-10-15

**Authors:** Adrienne Parsons, Esther Sauras Colon, Milos Spasic, Busem Binboga Kurt, Alexander Swarbrick, Rachel A. Freedman, Elizabeth A. Mittendorf, Peter van Galen, Sandra S. McAllister

**Affiliations:** 1 Division of Hematology, Department of Medicine, Brigham and Women’s Hospital, Boston, MA 02115, USA; 2 Department of Medicine, Harvard Medical School, Boston, MA 02115, USA; 3 Oncological Pathology and Bioinformatics Research Group, Hospital Verge de la Cinta, Institut d’Investigació Sanitària Pere Virgili, Universitat Rovira i Virgili, Tortosa, Tarragona, Spain.; 4 Division of Breast Surgery, Department of Surgery, Brigham and Women’s Hospital, Boston, MA 02115, USA; 5 Breast Oncology Program, Dana-Farber Brigham Cancer Center, Boston, MA, USA.; 6 Cancer Ecosystems Program, Garvan Institute of Medical Research, Darlinghurst, New South Wales, Australia; 7 St. Vincent’s Clinical School, Faculty of Medicine, University of New South Wales, Sydney, New South Wales, Australia; 8 Department of Medical Oncology, Dana-Farber Cancer Institute, Boston, MA 02115, USA; 9 Breast Cancer Program, Dana-Farber/Harvard Cancer Center, Boston, MA 02115, USA; 10 Broad Institute of Harvard and MIT, Cambridge, MA 02142, USA; 11 Harvard Stem Cell Institute, Cambridge, MA 02138, USA; 12 Ludwig Center at Harvard, Harvard Medical School, Boston, MA 02115, USA

## Abstract

Aging is associated with increased breast cancer risk and outcomes are worse for the oldest and youngest patients, regardless of subtype. It is not known how cells in the breast tumor microenvironment are impacted by age and how they might contribute to age-related disease pathology. Here, we discover age-associated differences in cell states and interactions in human estrogen receptor-positive (ER+) and triple-negative breast cancers (TNBC) using new computational analyses of existing single-cell gene expression data. Age-specific program enrichment (ASPEN) analysis reveals age-related changes, including increased tumor cell epithelial-mesenchymal transition, cancer-associated fibroblast inflammatory responses, and T cell stress responses and apoptosis in TNBC. ER+ breast cancer is dominated by increased cancer cell estrogen receptor 1 (*ESR1)* and luminal cell activity, reduced immune cell metabolism, and decreased vascular and extracellular matrix (ECM) remodeling with age. Cell interactome analysis reveals candidate signaling pathways that drive many of these cell states. This work lays a foundation for discovery of age-adapted therapeutic interventions for breast cancer.

Breast cancer is the second most commonly diagnosed cancer worldwide^1,2^. Relative to patients 55–64 years of age, both younger (<45) and older (>65) patients with early-stage disease have worse breast cancer-related outcomes, regardless of subtype, and older women fare the worst^3,4^. It is not known why breast cancer mortality rates are higher for the youngest and oldest women and the reasons are likely complex. Confounding our understanding is the fact that older women, despite accounting for the vast majority of breast cancer cases, are underrepresented in clinical trials^5,6^. Inclusion of women under the age of 40 in clinical trials is also rare due to the fact that they represent only ~7% of all breast cancer cases^7^. These deficits raise the question of whether trial results reflect real world outcomes.

Age at diagnosis affects prognosis differently based on molecular subtype^8–10^. Young women are more likely to develop more aggressive subtypes of breast cancer, such as triple-negative breast cancer (TNBC). Furthermore, young age is considered an independent risk factor for TNBC recurrence and death^7,11,12^. The incidence of all breast cancer subtypes increases with age, with hormone receptor-positive (HR+) disease increasing the most dramatically and thus representing the most prevalent subtype among older women^13^. These facts suggest there are age-related factors that underlie breast cancer initiation and progression.

Current breast cancer treatments are often tailored to the needs of different age populations due to tolerability, comorbidities, and variable toxicity^14–17^; however, very little is known about the relationship between age and treatment efficacy. We may therefore be missing opportunities for care and treatment of different breast cancer patient populations.

Results from prior studies, including our own, show that the breast cancer landscape is molecularly distinct with age^17,18^. Various prognostic “aging” signatures have been developed^19–22^; however, these signatures were derived from bulk data and are subtype agnostic. We reasoned that a deeper understanding of the age-associated molecular and biological programs defining breast cancer at cell-type resolution that also considers the well-documented biological and prognostic differences observed across breast cancer subtypes could provide a foundation for developing age-specific treatments, which may be necessary to improve outcomes for all patients.

In this Analysis, we develop a comprehensive computational framework for understanding cell-specific age-associated changes in gene expression and intercellular interactions within the tumor microenvironment of TNBC and estrogen receptor-positive (ER+) breast cancers. Our results establish that age is a strong driver of microenvironment heterogeneity, and that tumor-associated epithelial, immune, and stromal cell types are biologically distinct with age in a breast cancer subtype-dependent manner. Collectively, our results offer new insights into age-related functional programs, suggesting that breast cancer therapies could be improved by tailoring them to age-related molecular features.

## Results

### Age-related gene expression and functional gene set enrichment in TNBC and ER+ breast cancer

To characterize the age-related molecular landscape of breast cancer, we analyzed bulk gene expression in age-stratified tumors from patients with TNBC and ER+/HER2− (ER+) subtypes. We used the Molecular Taxonomy of Breast Cancer International Consortium (METABRIC) bulk gene expression database to identify differentially expressed genes (DEGs) between patients <45 years (“younger”) and >65 years (“older”) at diagnosis with stage I-III disease (Supplementary Table 1).

**Figure 1 |:**
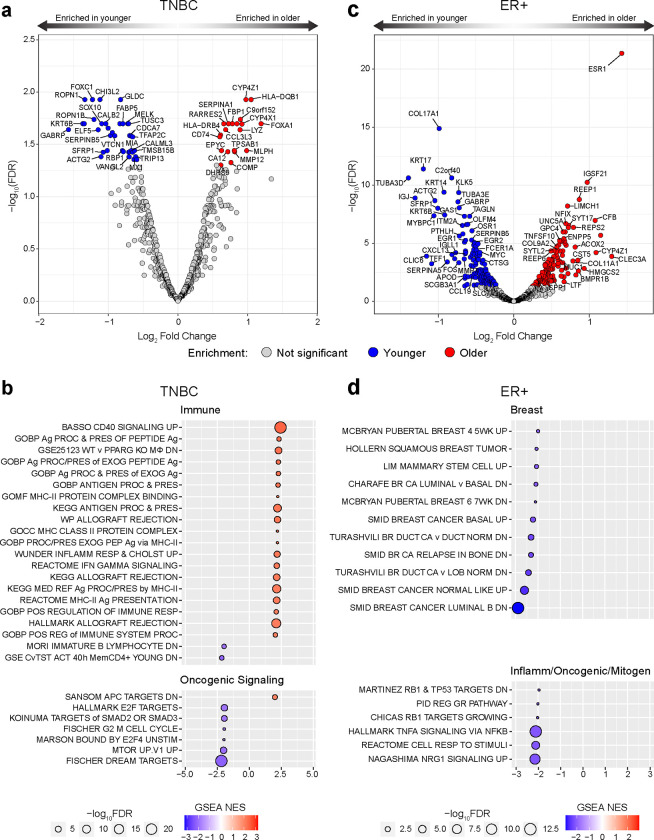
Age-related differentially expressed genes and functional gene set enrichments in TNBC and ER+ breast cancer. **a, c,** Volcano plots showing log2fold change for high variance genes (by standard deviation) in tumors from patients with TNBC (**a**) and ER+ breast cancer (**c**), comparing the age groups < 45 years and > 65 years from METABRIC. Plots show log_2_fold change difference on the x-axis and −log_10_FDR on the y-axis. Red colored dots represent genes enriched in the >65 age group; blue colored dots are genes enriched in the <45 age group; false discovery rate <0.05 (−log_10_FDR > 0.1.301). n=50 TNBC <45; n=63 TNBC >65; n=86 ER+ <45; n=386 ER+ >65. **b, d,** Results of age-stratified gene set enrichment analysis (GSEA) of highly variable genes ranked by log_2_fold difference from **a** and **c** in TNBC (**b**) and ER+ breast cancer (**d**). Pathways are grouped by biological similarity. Red fill color indicates enrichment in the > 65 age group; blue indicates enrichment in the < 45 age group. Circle size is proportional to relative −log_10_(FDR) for the enrichment, and color depth represents magnitude of normalized enrichment score (NES), according to indicated scales.

For TNBC, 38 DEGs were significantly enriched in tumors from younger patients and 20 in tumors from older patients when assessing the genes with the highest overall variance across the entire TNBC cohort (Fig. 1a, Supplementary Table 2). Gene set enrichment analysis (GSEA) of these highest variance genes yielded pathways associated with immune processes, nearly all of which were enriched in the older patients; these included antigen presentation and processing (particularly via MHC-II), inflammation response, and interferon gamma (IFNγ) signaling (Fig. 1b Supplementary Fig. 1a, Supplementary Table 3). In the younger TNBC cohort, gene sets involved in cell cycle and oncogenic signaling pathways were significantly enriched (Fig. 1b).

In the ER+ tumors, assessing the genes with highest overall variance across all ER+ patients yielded 135 DEGs that were significantly enriched in the older group and 139 in the younger group (Fig. 1c, Supplementary Table 2). In agreement with a prior report^17^, *ESR1* (encoding the estrogen receptor 1) was highly enriched in the older cohort (Fig. 1c). GSEA showed that all the differentially enriched pathways in ER+ were enriched in the younger cohort and included gene sets related to breast biology, breast cancer molecular subtype, and mitogenic stimuli (Fig. 1d, Supplementary Fig. 1b, Supplementary Table 3). Unlike TNBC, ER+ breast cancers did not display age-stratified immune responses, apart from tumor necrosis factor alpha (TNFα) signaling, which was enriched in the younger cohort (Fig. 1d, Supplementary Table 3).

These results revealed age-stratified molecular landscapes that are distinct between TNBC and ER+ breast cancer. Nevertheless, these bulk transcriptomic analyses did not capture the cell-specific context required for obtaining actionable insights.

### Development of a single-cell Age-Specific Program ENrichment (ASPEN) analysis method

**Figure 2 |:**
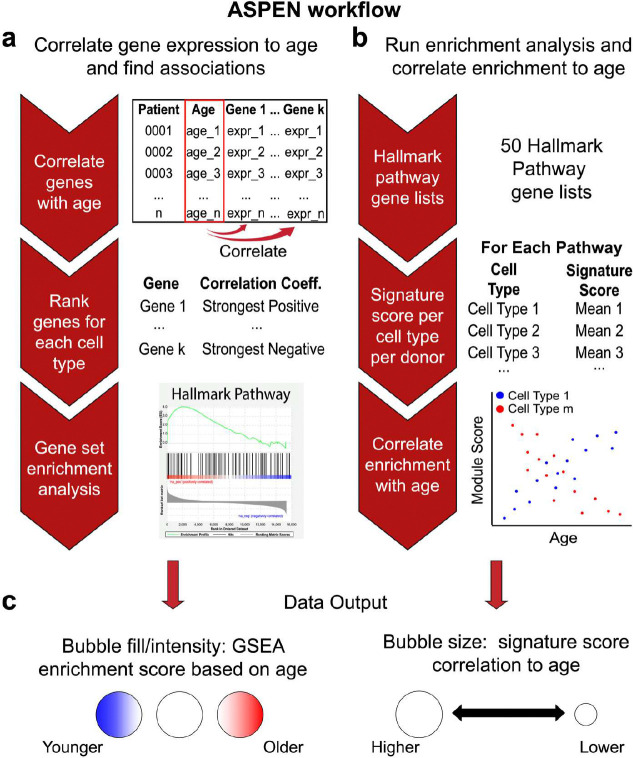
Development of a single-cell Age-Specific Program ENrichment (ASPEN) analysis pipeline. ASPEN relies on adaptations of gene set enrichment analysis (GSEA) in parallel assessments to correlate gene expression-based enrichment of functional pathways to age. **a**, The average gene expression per cell type is matched to donor age and a correlation coefficient for each gene is calculated. The genes with nonzero coefficients are then ranked by their correlation coefficients, and GSEA is performed using select gene sets of choice. **b** Concurrently, the gene sets are used to assign a signature score to every cell in the single-cell dataset using Seurat v4 commands. Following scoring, the mean signature score for each gene set is calculated per cell type per donor. These mean values are then correlated to donor age. **c**, The resulting normalized enrichment scores (NES) from a are then plotted as data point color for each cell type/pathway combination, with red indicating statistically significant enrichment in older donors, blue indicating statistically significant enrichment in younger donors, and white indicating a failure to achieve statistical significance. Depth of color is related to magnitude of enrichment. Irrespective of correlation direction (coefficient < 0 or coefficient > 0) in **b**, the magnitude of the correlation of signature score to age is visualized as the size of the data point for each cell type/pathway combination, with point size being proportional to the magnitude of correlation (larger circle = more strongly correlated or anti-correlated).

To leverage the resolution afforded by single-cell genomics for deeper insights into age-related breast cancer transcriptomes, we developed a single-cell RNA-seq data analysis platform, termed Age-Specific Program ENrichment (ASPEN), that identifies gene sets (e.g., MSigDB Pathways) that correlate with age across individual cell populations. ASPEN incorporates two parallel methods (Fig. 2). First, for each annotated cell type, expressed genes are ranked by their strength of correlation with age and then GSEA is performed (Fig. 2a). Second, a signature scoring algorithm is used to generate a gene set score per cell type per donor followed by correlation of the average signature score per cell type to age^23^ (Fig. 2b). Those two measures are visualized with a bubble plot in which the bubble fill color represents the GSEA normalized enrichment score (NES), and the bubble size represents the strength of signature correlation (Fig. 2c). Overall, this method is optimized to highlight the strongest age-associated differences in gene expression programs for individual cell types.

### Cell-specific Age-Related Programs (ARPs) show global enrichment in TNBC and reduction in ER+

In order to elucidate cell-specific gene expression programs that correlate with age in breast cancer, we used the single-cell and spatially resolved human breast cancer atlas^24^. The dataset includes 10 TNBC samples (n=42,512 total cells, average age 55.3 years, age range 35–73) and 11 ER+ samples (n=38,241 total cells, average age 60.9 years, age range 42–88). There were insufficient numbers of cells from HER2+ samples across the age spectrum for these analyses.

We first examined the abundance of the 8 major cell populations identified in the atlas with age (“celltype_major”: cancer epithelium, normal epithelium, cancer-associated fibroblasts (CAFs), myeloid cells, T cells, B cells, endothelium, and perivascular-like (PVL) cells), and their composition based on 29 annotated functional cell subpopulations also noted in the atlas (i.e., “celltype_minor” as a proportion of its respective “celltype_major”)^24^. In TNBC, the proportion of myeloid cells increased (R^2^ = 0.67, p = 0.034) and CAFs decreased (R^2^ = −0.66, p = 0.037) with age (data not shown), and an age-related decrease in CD4+ T cells as a percentage of all T cells approached significance (R^2^ = −0.62, p = 0.054; Supplementary Fig. 2a, b). In the ER+ tumors, the abundance of major populations did not change with age (data not shown), yet the proportion of inflammatory CAFs (iCAFs) (R^2^ = −0.71, p=0.014), and differentiated PVL cells (R^2^ = −0.59, p=0.055) had negative relationships with age. Positive correlations with myofibroblast-like CAFs (myCAFs) (R^2^ = 0.71, p=0.014) and immature PVL cells (R^2^ = 0.63, p=0.039) were also identified (Supplementary Fig. 3a, b). We also noticed a trend toward increased luminal A and decreased luminal B cell abundance in the ER+ tumors with age, consistent with prior reports of luminal A predominance in older ER+ breast cancer patients^13,25^

**Figure 3 |:**
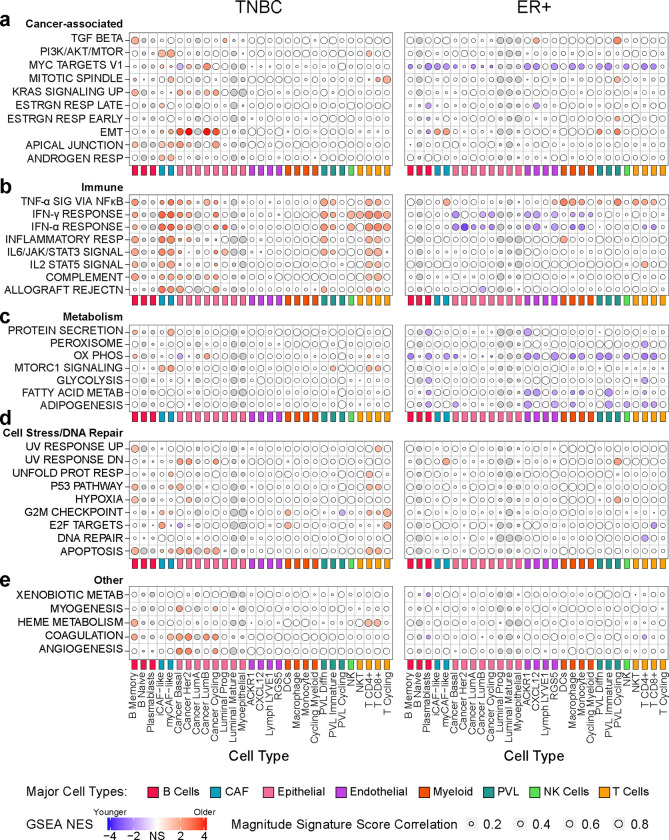
Cell-specific age-related programs (ARPs) in TNBC and ER+ breast cancer. Results from ASPEN analysis of the breast cancer single-cell RNA-seq atlas dataset^24^ and Hallmark gene sets (Human MSigDB) yielding cell-specific age-related programs (ARPs) in TNBC (left) ER+ breast cancer (right).^24^ The 29 minor cell types (color coded by indicated major cell type groups) are represented on x-axes and indicated Hallmark pathways on y-axes. ARPs were manually grouped into biologically similar processes, including cancer-associated (**a**), immune-related (**b**), metabolism (**c**), cell stress/DNA repair (d), and others (**e**). A given cell type must have been present in >50% of donors for that cell type to be correlated to donor age; otherwise, it was excluded from analysis. Donors with a cell count of 0 for a given cell type were excluded from analysis of that cell type. Bubble color indicates normalized enrichment score (NES) of age-associated GSEA analysis (Fig. 2a), with deeper color indicating greater enrichment. Red indicates statistically significant enrichment (adjusted p < 0.05) in older donors; blue indicates statistically significant enrichment (adjusted p < 0.05) in younger donors; white indicates a failure to achieve statistical significance; gray indicates cell types that were not assessed because they were present in < 50% of the donors. Bubble size indicates magnitude of enrichment score correlation to age (Fig. 2b); larger bubbles indicate stronger correlation or anti-correlation. NS = not significant, TNF = tumor necrosis factor, SIG = signaling, IFN = Interferon, RESP = response, SIGNAL = Signaling, REJECTN = rejection, OX PHOS = oxidative phosphorylation, METAB = metabolism, TGF = transforming growth factor, ESTRGN = estrogen, EMT = epithelial to mesenchymal transition, DN = down, UNFOLD PROT RESP = Unfolded Protein Response, CAF = cancer associated fibroblast, PVL = perivascular-like cells.

We then applied ASPEN to identify age-related programs (ARPs), defined as gene expression sets (e.g. Hallmark pathways from MsigDB) that correlate with age. Global analysis of normalized enrichment scores (NES) from each of the 29 minor cell populations revealed that most ARPs increased with age in TNBC and decreased with age in ER+ breast cancer (Supplementary Fig. 4), consistent with results from the METABRIC analysis (Fig. 1b, d). Enrichment patterns were unique to each breast cancer subtype (Figure 3), a finding that was conserved when we applied ASPEN to the 49 most granular annotated cell subtypes (“celltype_subset”, Supplementary Fig. 5)^24^. Details of the ASPEN results for each breast cancer subtype follow in subsequent sections.

To evaluate senescence as a potential driver of age-associated gene expression changes, we applied ASPEN to published senescence signatures. While some signatures were enriched in older patients (e.g. CAFs in TNBC), senescence ARPs were not observed in other cell populations and notably mostly absent from ER+ breast cancer (Supplementary Fig. 6). These initial observations indicated that although the proportions of most tumor-associated cell types did not change significantly with age, their respective transcriptional programs did.

### Cell-specific enrichments of epithelial-mesenchymal transition, immune responses, and stress responses with age in TNBC

We inspected some of the ARPs to gain deeper insights into cell-specific cellular activity within the tumor microenvironment (TME) with age.

In TNBC, epithelial-mesenchymal transition (EMT) in cancer epithelial cell subpopulations represented the strongest overall enrichment with increased age (Fig. 3a, Supplementary Table 4). EMT is a cellular program that confers enhanced tumor-initiating capacity, invasion, and metastatic potential^26,27^. In the basal cancer cells, which are a dominant cancer epithelial cell type in these tumors (Supplemental Fig. 2a), enrichment of EMT was concurrent with increased immune response, K-Ras signaling, cellular stress responses, and angiogenesis, and decreased oxidative phosphorylation, myc targets, and E2F targets with age (Fig. 3a–e).

In agreement with enrichment of immune pathways with increased age from the METABRIC analysis (Fig. 1b), immune function and inflammation were positively correlated with age in several cell populations, including CD4+ T cells, CD8+ T cells, iCAFs and myCAFs (Fig. 3b, Supplementary Table 4). Of the immune programs, the strongest ARPs (i.e., those with the highest NES) occurred in both subsets of CAFs, whereby interferon (IFNα and IFNγ) response pathways were increased with age (Fig. 3b, Supplementary Table 4). CD4+ and CD8+ T cells displayed elevated stress responses and apoptosis with age (Fig. 3d). Despite the increased numbers of myeloid cells with age, monocyte/macrophage populations displayed no ARPs (Fig. 3a–e).

These results established ARPs for specific cell types in TNBC. The age-associated changes in some cell types (T cells, CAFs) were reflected in altered gene expression, whereas other cell types (myeloid) predominantly show altered abundance. With increasing age, TNBC is dominated by cancer cells with an EMT phenotype and an inflamed microenvironment in which T cells and CAFs display responses to cellular stress and immune stimuli.

### Cell-specific reductions in metabolism, myc targets, and interferon responses with age in ER+ breast cancer

Unlike TNBC, and consistent with the METABRIC analysis (Fig. 1c, d), the majority of ARPs in ER+ breast tumors were enriched in the younger cohort (Fig. 3, Supplementary Figs. 4, 5). The only significant ARPs in the epithelial subpopulations were high interferon responses and myc targets in the youngest donors (Fig. 3a, b, Supplementary Table 4). There were no estrogen response ARPs in the tumor epithelial cells (Fig. 3a) despite the shift toward luminal A and high *ESR1* expression observed in the older METABRIC cohort (Fig. 1c and Supplementary Fig. 3).

Unlike TNBC, metabolic processes were significantly enriched in the youngest ER+ donors, particularly in the vasculature, plasmablasts, CD4+ and CD8+ T cells (Fig. 3c, Supplementary Table 4). In other words, these cell populations were less metabolically active in the older ER+ cohort.

Unlike the myeloid compartment of TNBC, which exhibited no ARPs, the myeloid cells in ER+ tumors appeared to be programmed differently with age. Specifically, TNFα signaling significantly correlated with increasing age, while interferon responses decreased with age (Fig. 3b, Supplementary Table 4). Monocytes/macrophages were also significantly less metabolically active with age (Fig. 3c). These results suggested type I inflammatory responses in the younger cohort and a tumor-promoting inflammatory phenotype^28^ in the older cohort in ER+ tumors.

ASPEN revealed significant differences in T cell populations with age in the ER+ tumors, which were similar for CD4+ and CD8+ T cells. Specifically, TNFα and interleukin-2 (IL2) signaling increased while IFNα responses, myc targets, and, as already mentioned, metabolism, decreased with age (Fig. 3 a–c). These findings were consistent when evaluating the specific cell subpopulations (“celltype_subset”; Supplementary Fig. 5).

Collectively, the cell-specific ARPs in ER+ tumors indicated enrichment of tumor-supportive inflammatory activity in myeloid cells, reductions in metabolically active endothelium, and attenuated interferon responses in cancer cells with age. The ARPs in CD4+ and CD8+ T cells suggested quiescence, exhaustion, and metabolic dysfunction with increasing age.

### Age-differential cellular interactomes in TNBC and ER+

Having identified key cell-specific age-related transcriptional programs, we investigated whether cell-cell interactions differ with age by employing CellChat. CellChat integrates the expression of ligands, receptors, cofactors, multimeric receptor-ligand complexes, soluble agonists and antagonists, and stimulatory and inhibitory membrane-bound coreceptors as well as abundance of each cell type to infer the likelihood of a specific ligand-receptor pair interaction between specific cells^29^. For these analyses, we stratified the single-cell data^24^ by age (≤55 and >55) for both breast cancer subtypes.

**Figure 4 |:**
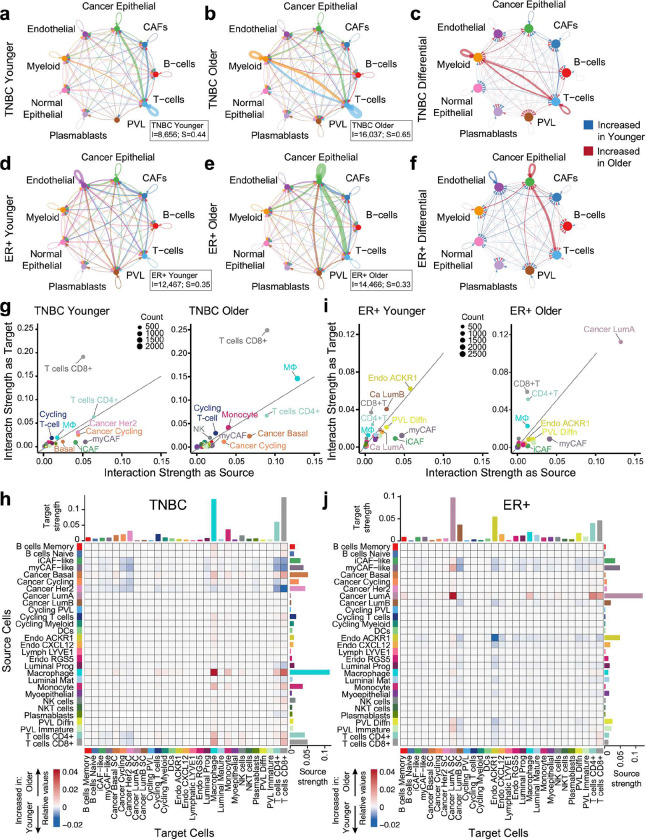
Age-related cell-cell interactions in TNBC and ER+ breast cancer. **a-f**, Circle plot visualizations of the predicted homotypic and heterotypic interaction strength between major cell types in TNBC (**a-c**) and ER+ breast cancer (**d-e**) tumors from the single-cell RNA-seq atlas^24^ using CellChat v2 analysis. Circle plots are shown for patients ≤55 (younger, **a, d**), patients >55 (older, **b, e**), and the differential between age groups (**c, f**) for each subtype. TNBC ≤55 years (n=6, N=20,591 cells), TNBC >55 years (n=4, N=20,203 cells), ER+ ≤55 years (n=6, N=21,735 cells), ER+ >55 years (n=5, N=15,344 cells). Indicated cell types are represented by colored nodes; edge colors in **a**, **b**, **d**, e correspond to the source cell type; edge colors in **c**, **f** indicate stronger interaction strength in the older cohort (red) or the younger cohort (blue). Line thicknesses are proportional to the strength of interaction between given cells. Boxed insets in **a, b, d, e** indicate total number of interactions (I) and total interaction strength (S) for each cohort. **g, i**, Scatter plots representing the interaction strengths of each of the 29 minor cell types as a signaling source (x axes) and target (y axes) for indicated age cohorts in TNBC (**g**) and ER+ breast cancer (**i**). Dot sizes represent the number of interactions (count) for each cell type. **h, j**, Heat maps representing differential interaction strengths between each indicated target cell (x axes) and source cell (y axes) for TNBC (**h**) and ER+ breast cancer (**j**). Color scale is based on the differential interaction strength; shades of red indicate stronger interaction in the older cohort; shades of blue are stronger in the younger cohort. Bar plots at top of heat maps correspond to the absolute sum of differential incoming interaction strength for each cell type; bar plots at right of the heat maps correspond to the absolute sum of outgoing interaction strength for each cell type. Cell type color annotations are consistent throughout **g-j**.

In TNBC, the older cohort exhibited a 1.85-fold increase in total cell-cell interactions and a 1.48-fold increase in interaction strength (Fig. 4a, b; Supplementary Table 6). Both younger and older TNBC cohorts showed strong interactions between cancer epithelial cells and T cells, and among T cells themselves (Fig. 4a, b). Age impacted the TNBC interactome whereby the older cohort was dominated by bidirectional myeloid:T cell communication and the younger cohort was enriched for CAF interactions with T cells and cancer epithelium (Fig. 4c; Supplementary Table 6). These results align with METABRIC and ASPEN analyses, which highlighted immune related ARPs (Figs. 1a, b, 3b).

In ER+ breast cancer, the older cohort had a 1.16-fold increase in total interactions but a 1.06-fold decrease in interaction strength (Fig. 4d, e; Supplementary Table 6). Both age groups displayed strong CAFs:cancer epithelial interactions (Fig. 4d, e). Interactions between cancer epithelial cells and both myeloid and T cells dominated the older cohort, while interactions within the vascular compartment (endothelium and PVLs; Fig. 4f) were enriched in the younger cohort, consistent with increased metabolic activity observed earlier (Fig. 3c).

To refine our understanding of which cells accounted for the most significant age-related differences, we examined the 29 minor cell subpopulations. This revealed numerous age-stratified interactions, and we describe only the most predominant of those.

In TNBC, cancer basal cells enriched their communication with macrophages, CD4+, and CD8+ T cells (Fig. 4g, h). Despite the rise in CAF-specific ARPs with age (Fig. 3), CAF interactions were stronger in younger TNBC patients, particularly through myCAF and iCAF signaling to CD4+ and CD8+ T cells (Fig. 4g, h, Supplementary Table 6). Macrophages exhibited the most significant age-related interaction changes, marked by homotypic interactions and increased communication with cancer basal cells, monocytes, CD4+ T cells, and CD8+ T cells (Fig. 4g, h), despite their lack of ARPs (Fig. 3).

In TNBC, increased macrophage:T cell interactions with age, along with signals of enhanced MHC-II presentation from the METABRIC bulk analysis (Fig. 1b), led us to examine cell-specific MHC class II expression. While professional antigen-presenting cells (APCs) accounted for most MHC-II (HLA) gene expression, age-biased expression was driven by CAFs, vascular cells, and cancer cells in the older patients (Supplementary Fig. 7a), suggesting IFNγ^30^ exposure and aligning with enriched interferon response genes in these cells (Fig. 3b).

In ER+ breast cancer, ACKR1+ endothelial cells had 15-fold increased homotypic interactions and significantly more interactions with various cell populations in the younger cohort (Fig. 4i, j, Supplementary Table 6), aligning with their enhanced protein secretion, and metabolic activity ARPs in younger patients (Fig. 3b). ACKR1 modulates innate immunity by trafficking chemokines^31^; hence, their enhanced interferon response ARPs suggests stronger immune modulation in the younger cohort. In addition to their increased abundance in the younger cohort (Supplementary Fig. 3a), iCAFs were a dominant signaling source in the younger cohort, whereas myCAFs were increased in abundance (Supplementary Fig. 3a) and had stronger interactions with luminal A cells with age (Fig. 4i, j). Cancer luminal A cells exhibited the most dramatic age-related interaction changes, with significant increases in autocrine and immune cell interactions with age (Fig. 4i, j, Supplementary Table 6).

The significant activity of the cancer epithelium in ER+ breast cancer, along with elevated *ESR1* with age in METABRIC (Fig. 1c), prompted us to examine cell-specific *ESR1* expression. *ESR1* was significantly higher in luminal A and luminal B cancer cells in older patients (Supplementary Fig. 7b), although, as mentioned earlier, estrogen response gene sets did not correlate with age in these cells (Fig. 3a).

Collectively, these results revealed distinct intercellular interactions that occur in an age-biased manner in TNBC and ER+ breast cancer. Although our study is not powered to analyze the spatial transcriptomic data from these tumors by age, prior analysis^24^ confirms the co-localization of the cell types we identified through these analyses.

### Identifying age-stratified signaling networks in TNBC and ER+

We next explored the molecular basis for age-biased cell-cell interactions in TNBC and ER+ breast cancer. Evaluating the 3,234 annotated ligand-receptor pairs in the CellChat database across each of the 29 minor cell types for each breast cancer subtype risked producing an overwhelming number of signaling nodes. Therefore, we developed regression-based selection criteria to identify subsets of cells on which to focus and the most prominent signaling interactions between them (see Methods).

Specific ligand-receptor pairs are categorized into general “signaling pathways”^29^ and we use the term “signaling interaction” to denote signaling pathways predicted to be activated between specific cell types and “signaling node” to refer to the specific ligand-receptor pair(s) activated between cells. Results for each breast cancer subtype are described in the following sections.

### Age-associated signaling network in TNBC

The selection criteria yielded 7 cell types for TNBC: iCAF, myCAF, basal cancer cells, macrophages, monocytes, CD4+, and CD8+ T cells (7×7=49 possible source/target cell combinations). We used the CellChat *rankNet* function to calculate scaled interaction weights across these 49 combinations, yielding 650 signaling interactions comprising 71 different signaling pathways (Supplementary Fig. 8). 483 (74%) of these had higher probability values in the older cohort, 307 signaling interactions were exclusive to the older cohort, and 48 were exclusive to the younger cohort (Supplementary Fig. 8). Nine signaling pathways, supported by 43 ligand-receptor pairs, were identified as the most dominant across all selected cells in one or both age cohorts (Supplementary Table 7).

**Figure 5 |:**
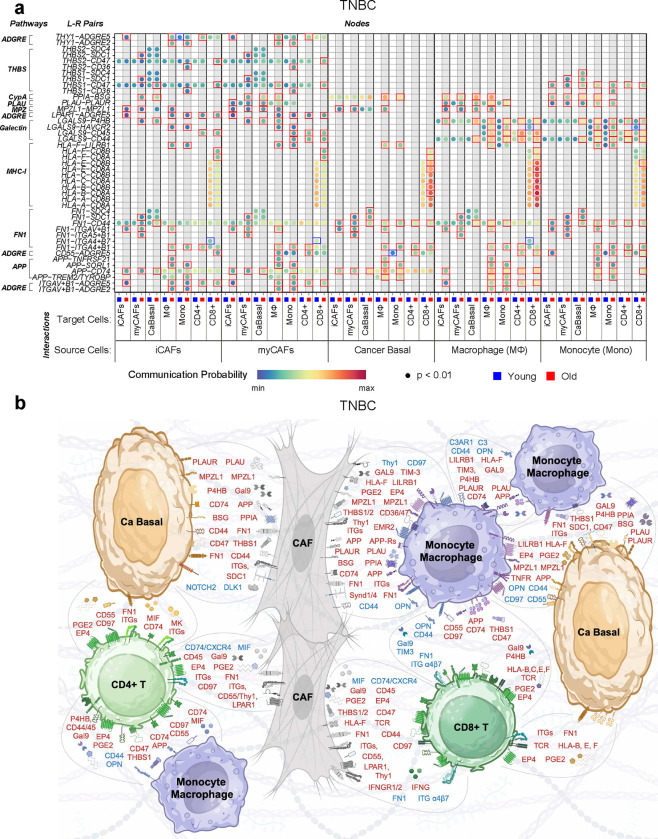
Age-associated signaling network in TNBC. **a,** Bubble plots representing the communication probability for each indicated ligand-receptor pair between indicated source:target cells for each age cohort (See Methods, Supplementary Table 7, and Supplementary Fig. 8). Rows depict the ligand-receptor pairs and signaling pathways; columns depict specific source-target cell interactions for the ≤55 cohort (blue) or >55 cohort (red). Communication probabilities are represented by a color scale, with minimum values colored deep blue, increasing values depicted as green, then yellow, then orange, and maximum values as deep red. Each bubble represents a signaling node predicted to be active with FDR value < 0.01 through CellChat probability calculations^29^. Colored boxes around bubbles indicate signaling nodes that had probabilities detected at p < 0.05 in at least one age group and the difference in that probability was at least 1.2-fold greater in either the younger (blue boxes) or older (red boxes) cohort. **b**, Schematic representation of the signaling nodes in **a** and additional signaling nodes of interest following manual curation of specific cell-cell interactions (Supplementary Fig. 8, Supplementary Table 7). For clarity of representation, data were combined for monocytes/macrophages and iCAFs/myCAFs. Blue text indicates enrichment in the ≤55 age group; red text indicates enrichment in the >55 age group.

The monocyte/macrophage-derived galectin 9 (Gal9, *LGALS9*) signaling interaction showed the greatest difference between TNBC age groups, being elevated in older patients (Fig. 5a). Gal9 signaling via prolyl 4-hydroxylase beta (*P4HB*) is linked to EMT promotion and age-related cancers^32^. Signaling nodes between CAF- and monocyte/macrophage-derived Gal9 and P4HB on cancer basal cells were exclusive to the older cohort (Fig. 5a, b; Supplementary Fig. 8). Gal9-CD44 signaling increased with age, which promotes regulatory T cell (Treg) function and CD8+ T cell death^33^. CAF− and monocyte/macrophage-derived Gal9 signaling to CD4+ and CD8+ T cells was higher in the older cohort (Fig. 5a, b). This result aligns with ASPEN analysis that showed increased T cell apoptosis with age (Fig. 3d).

Given that the interaction between macrophages and T cells represented one of the strongest cell:cell interactions in older patients (Fig. 4c), we manually curated the specific signaling nodes for these cell types for further investigation. We observed additional immunosuppressive factors (e.g., PGE2 and MIF) expressed by myeloid cells in the older cohort (Fig. 5b, Supplementary Fig. 8d, e, Supplementary Table 7).

Plasminogen activator, urokinase (*PLAU*) and cyclophilin A (*PPIA*), which promote EMT in tumor cells and fibrosis in CAFs^34–36^, were enriched in signaling nodes between myCAFs:cancer cells and monocyte/macrophages:cancer cells in the older cohort (Fig. 5a, b), supporting the EMT ARPs previously observed (Fig. 3). In this cohort, CD55 expressed by CAFs, basal cancer cells, and monocyte/macrophages was predicted to interact with CD97 (*ADGRE5*) on CD4+ T cells, which promotes Treg development^37^. Immune-suppressive signaling nodes^38,39^ between the class I molecule, HLA-F, on both CAFs and cancer basal cells with the inhibitory receptor, leukocyte immunoglobulin like receptor B1 (*LILRB1*), on monocyte/macrophages were also noted in older TNBC patients.

Three signaling interactions were prominent CAFs in the older cohort, involving the nodes THY-1 cell surface antigen (CD90, *THY1*), lysophosphatidic acid receptor 1 (*LPAR1*), and thrombospondins 1 and 2 (*THBS1, THBS2*) (Fig. 5a, b). LPAR1 signaling via CD97 (*ADGRE5*) promotes fibrosis and chemoresistance in TNBC^40^. THY1-positive CAFs, annotated as iCAFs in the atlas^24^ are known to suppress T-cell function^41^, promote Treg recruitment ^42^ and were associated with poor outcome in glioblastoma^43^. Elevated THBS1 signaling, particularly via syndecan-1 (*SDC1*), which was observed in CAFs in the older cohort (Fig. 5a, b), aids cancer cell motility^44^ and is associated with reduced survival in breast cancer^45^.

Expression of the CD8+ T cell receptor, concomitant with HLA genes associated with MHC-I on cancer basal cells, was higher in the older cohort (Fig. 5a, b), although peptide presentation could not be deciphered. Nevertheless, immunosuppressive signaling interactions were also elevated; for example, immunosuppressive prostaglandin E2 (PGE2), macrophage inhibitory factor (MIF), and midkine (MK) were elevated between cancer basal cells and CD4 and CD8 T cells in the older cohort (Fig. 5b, Supplementary Fig. 8c).

Confirming immune modulatory ARPs in CAFs and cancer cells (Fig. 3b), type 2 interferon (IFN-γ) signaling from CD8+ T cells to iCAFs, myCAFs, and cancer basal cells was exclusive to the older cohort (Fig. 5b, Supplementary Fig. 8f). Exploring the potential source(s) of enriched antigen processing and presentation via MHC-II in the older cohorts from the METABRIC and ASPEN analyses, signaling nodes related to MHC-II presentation by cancer basal cells, myCAF, and iCAF to monocytes and macrophages were elevated in the older cohort (Fig. 5b, Supplementary Fig. 8a-e), confirming our earlier observation of various class II molecules on “non-professional” antigen presenting cells (Supplementary Fig. 7a).

Only a few of the signaling interactions were exclusive to or higher in the younger TNBC cohort. For example, a signaling node between monocyte/macrophage-derived Gal9 and the checkpoint protein TIM3 (*HAVCR2*) on CD8+ T cells, which suppresses antitumor immunity^46^ and induces CD8+ T cell death^47^, was elevated in the younger cohort (Fig. 5a, b). Macrophage-derived osteopontin (secreted phosphoprotein 1, *SPP1*) signaling to various cells exclusively in the younger cohort (Fig. 5b, Supplementary Fig. 8d) is consistent with promotion of breast cancer progression and chemoresistance^48–51^. Enriched fibronectin 1 (*FN1*) engagement of integrins α4/β7 on CD8+ T cells suggested regulation of T cell migration and T cell receptor activity in the younger cohort (Fig. 5a, b). Finally, we noted that CAF signaling to lymphocytes (CD8+ and CD4+) and myeloid cells (monocytes and macrophages) via MIF occurred exclusively in the younger cohort (Fig. 5b, Supplementary Fig. 8a, b). Hence, while certain signaling pathways, such as Gal9 and MIF, were activated broadly across the TME in the older cohort, they also played a role in specific cell-cell interactions in the younger cohort.

### Age-associated signaling network in ER+ breast cancer

For ER+ breast cancer, the selection criteria revealed 8 cell types: iCAF, myCAF, cancer luminal A, macrophages, ACKR1+ endothelial cells, differentiated PVLs, CD4+ T cells, and CD8+ T cells (8×8=64 possible source/target cell combinations). Across these 64 combinations, *rankNet* analysis yielded 745 signaling interactions comprising 84 signaling pathways (Supplementary Fig. 9). Of those interactions, 411 (55%) were more prevalent in the younger cohort, while 334 were more prevalent in the older cohort. The younger cohort had 166 unique interactions while 186 were unique to the older cohort (Supplementary Fig. 9). Hence, unlike TNBC, in which the balance of the strongest age-dependent signaling interactions tipped in favor of the older cohort, interactions were more evenly balanced between older and younger cohorts in the ER+ breast cancers.

**Figure 6 |:**
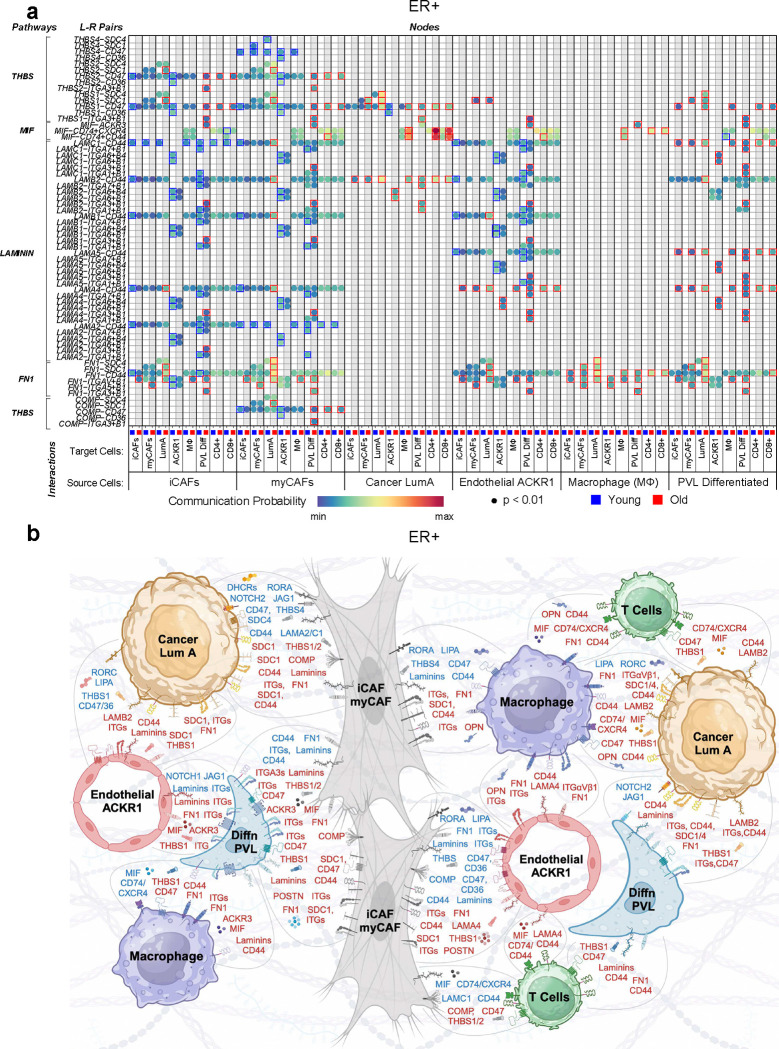
Age-associated signaling network in ER+ breast cancer. **a,** Bubble plots representing the communication probability for each indicated ligand-receptor pair between indicated source:target cells for each age cohort (See Methods, Supplementary Table 7, and Supplementary Fig. 9). Rows depict the ligand-receptor pairs and signaling pathways; columns depict specific source-target cell interactions for the ≤55 cohort (blue) or > 55 cohort (red). Communication probabilities are represented by a color scale, with minimum values colored deep blue, increasing values depicted as green, then yellow, then orange, and the maximum values as deep red. Each bubble represents a signaling node predicted to be active with FDR value < 0.01 in the CellChat probability calculation^29^. Colored boxes around bubbles indicate signaling nodes that were differentially enriched by at least 1.2- fold, in either the younger (blue boxes) or older (red boxes) cohort. **b,** Schematic representation of the signaling nodes in **a** and additional signaling nodes of interest following manual curation of specific cell-cell interactions (Supplementary Fig. 9, Supplementary Table 8). For clarity of representation, data were combined for iCAFs/myCAFs and CD8+/CD4+ T cells. Blue text indicates enrichment in the ≤55 age group; red text indicates enrichment in the >55 age group.

Adhesion proteins and extracellular matrix (ECM) engagement comprised the vast majority of the strongest signaling interactions across selected cell types in both age cohorts and included the non-collagenous glycoprotein family members, laminins, fibronectin (*FN1*), and thrombospondins (*THBS*) signaling through various heterodimeric integrin receptors (Fig. 6a, Supplementary Fig. 10, Supplementary Table 7). ECM engagement within the TME modulates cell proliferation, differentiation, adhesion, and migration, serves as a sink for cytokines, promotes angiogenesis and inflammation, and governs malignant progression ^52^. These various adhesion signaling nodes appeared to be both age-dependent and cell type-specific. For example, nearly all adhesion/ECM signaling nodes involving either cancer luminal A cells, differentiated PVLs, or macrophages were enriched in the older cohort, while those involving iCAFs, myCAFs, and ACKR1+ endothelial cells were mostly enriched in the younger cohort (Fig. 6a). T cells showed no engagement of these particular adhesion molecules (Supplementary Fig. 10), perhaps owing to the fact that ER+ breast cancer is often devoid of tumor-infiltrating lymphocytes^53^.

Periostin (*POSTN*) signaling represented the strongest, statistically significant signaling pathway (p<0.02, frequency = 12) and was highly enriched between CAFs and vascular cells (endothelium and PVLs) in the older cohort (Fig. 6b, Supplementary Table 7, Supplementary Fig. 9). Periostin is a matricellular protein that mediates fibrosis, angiogenesis and chemoresistance in cancer^54^.

We also observed age-biased expression and predicted interactions featuring various laminin subunits, which are the major non-collagenous components of the basement membrane^52^. For example, the cancer basal cells exclusively used the beta-2 subunit (*LAMB2*) in the older cohort (Fig. 6a). Moreover, ACKR1+ endothelial cells preferentially used the laminin subunit alpha-4 (*LAMA4*) to engage other cells in the older cohort, while using various other laminins to engage those same cells in the younger cohort (Fig. 6a). There also seemed to be aged-biased usage of integrin subunits, particularly the use of alpha-3/beta-1 by differentiated PVL cells in the older cohort (Fig. 6a).

Like TNBC, MIF signaling between cancer luminal A cells and CD4+ or CD8+ T cells via CD74 complexes was elevated in the older cohort (Fig. 6a, b, Supplementary Fig. 9). Tumor cell-derived MIF promotes expansion of Tregs (consistent with the IL-2 ARP we observed in CD4+ T cells via ASPEN; Fig. 3b) and inhibits CD8+ T cell activation^55^. We also note that osteopontin (*SPP1*)-expressing macrophages, which promote disease progression, communicated exclusively in the older cohort (Supplementary Fig. 9), in agreement with the METABRIC analysis, which revealed significantly higher expression of *SPP1* in the older cohort (Fig. 1c).

Notch signaling to cancer luminal A cells was enriched in the younger cohort, specifically from PVL cells and myCAFs (Fig. 6b, Supplementary Fig. 9, Supplementary Table 7). Notch plays a critical role in maintaining luminal progenitor cell fate in the breast^56^, thus in agreement with the luminal and stem cell pathways observed in the younger METABRIC cohort (Fig. 1d). Overexpression of notch receptors and ligands is correlated with TNBC progression and therapeutic resistance, but is less well described in ER+ breast cancer^56^.

### Age-related landscape of TNBC and ER+ breast cancer

**Figure 7 |:**
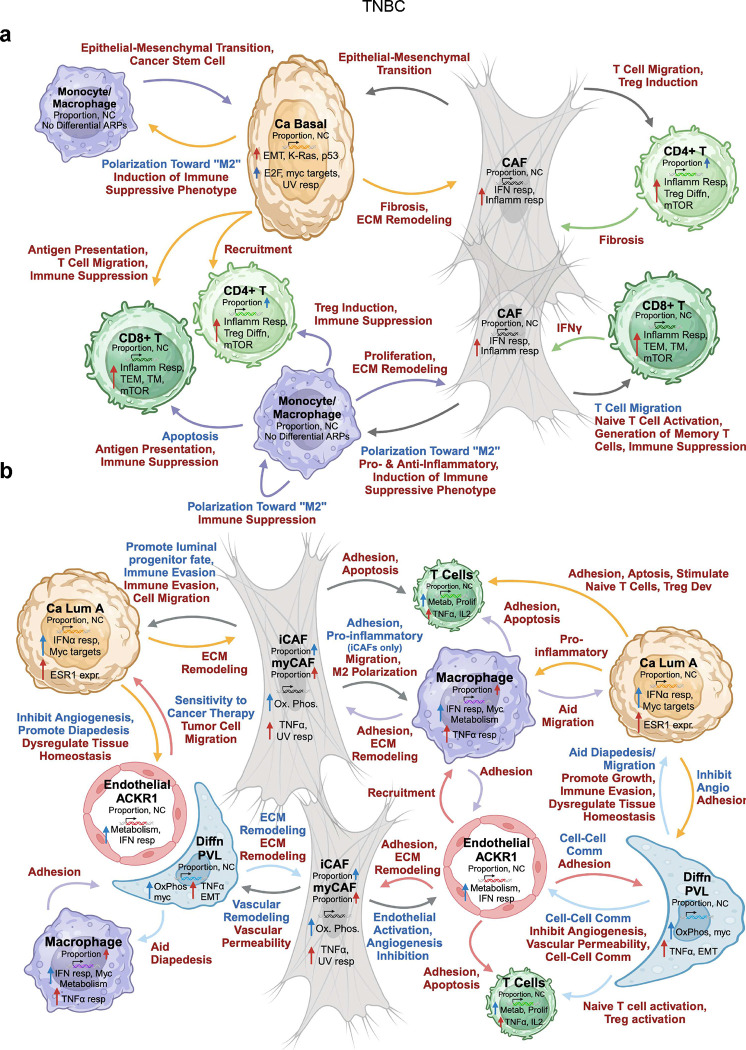
Working models of the age-related molecular landscapes of TNBC and ER+ breast cancer. **a, b,** Schematic depicts biologically distinct functions with age in the TNBC (**a**) and ER+ (**b**) breast tumor microenvironment. Selected cell types within the tumor microenvironment are shown with abundance, transcriptional (from METABRIC and ASPEN analyses), and communication (from CellChat analysis) differences with age. Arrows between cell types are colored to coincide with the source cell. Arrows within a cell type and all text depict enrichment in older (red) or younger (blue) patients.

Through detailed, manual integration of key cell-specific results from each dataset, we built a comprehensive age-related landscape of TNBC (Fig. 7a) and ER+ breast cancer (Fig. 7b).

In the older TNBC cohort, myeloid cells and CAFs interact with cancer basal cells through LGALS9/P4HB, PPIA/BSG, and PLAU/PLAUR ligand/receptor pairs to promote EMT and cell motility, as confirmed by the age-related increase in EMT ARPs. The mesenchymal-like cancer cells in turn impact the TME by: 1. presenting antigen to T cells, which aligns with the enrichment of inflammatory response and memory T cell ARPs; 2. promoting Treg development; 3. inducing CD8+ T cell death, consistent with apoptosis ARPs; and 4. generating an immune suppressive phenotype in monocyte/macrophages (Fig. 7a). Tumor cells in the older cohort also engage with CAFs to promote fibrosis and ECM remodeling, which is required for mesenchymal-like tumor cells to detach, leading to enhanced motility, invasion, metastasis, and chemoresistance^26^. CAFs were also observed to play a dominant role in modulating immune responses, as indicated by their inflammatory ARPs and signaling nodes that suppress T cell function and recruit Tregs. The increased MHC-II presentation by many cells within the older TME, consistent with METABRIC analysis, suggests exposure to IFNγ^30^, which is supported by the observed enrichment of interferon response ARPs, especially in CAFs. Given the dual role of IFN-γ in promoting anti-tumor immunity and mediating immune evasion ^57,58^, interferon signaling in CAFs warrants further investigation, particularly in the context of aging.

The absence of ARPs in monocyte/macrophages in TNBC aligns with our finding that these cells promote pro-tumorigenic and immunosuppressive phenotypes in both older and younger cohorts, but via different signaling nodes (Fig. 7a). In the younger TNBC cohorts, we observed basal cancer cells that were not enriched for EMT ARPs and CAFs with decreased inflammatory signaling compared to the older cohorts. Interactome analysis suggested these phenotypes were driven by macrophage polarization factors (Fig. 7a) that are linked to enhanced TNBC progression and metastasis^59^. The immunosuppressive profile of macrophages was shaped by different factors in younger and older TNBC cohorts (such as *SPP1* in younger), indicating that targeting immune suppressive macrophages may require age-stratified strategies.

In ER+ breast cancer, cell-specific ARPs indicated increased myeloid inflammatory activity, less metabolically active endothelium, attenuated cancer cell interferon responses, and CD4+/CD8+ T cell quiescence and metabolic dysfunction with age (Fig. 7b). These processes, including cell migration, vascular permeability, and immune trafficking, are influenced by ECM structure and alignment^60^. Signaling nodes in both age groups involved adhesion and ECM interactions, with different nodes active between cohorts, suggesting age-biased tissue remodeling as a key driver of these cell-specific ARPs in ER+ breast cancer (Fig. 7b).

In the younger ER+ breast cancers, increased ECM and vascular remodeling occurred via interactions between CAFs, endothelial ACKR1 cells, and differentiated PVL cells. The fact that ACKR1+ endothelial cells, which are involved in chemokine trafficking in innate immunity, were the most metabolically active cells in the younger ER+ cohort, together with their enriched signaling nodes with PVLs, suggests an important immunomodulatory role within the younger ER+ tumor microenvironment (Fig 7b).

The older ER+ breast cancers were enriched with myCAFs. When taken together with their ARPs and active signaling nodes using ligands, such as periostin, the results suggest CAFs as drivers of increased desmoplasia, chemoresistance, and promotion of cancer cell invasion with age in ER+ breast cancer.

Though ER+ tumors are characterized as immunologically “cold”^53^, age-related differences in immunogenicity were noted. Older tumors showed increased MIF activity, potentially attenuating eradication by cytotoxic T lymphocytes, as observed in lymphoma^61^. Furthermore, higher CD47 signaling in older T cells, as observed in melanoma models^62^, suggests reduced T cell cytotoxicity. Increased TNFα signaling in older tumors might also contribute to reduced immunogenicity through heightened inflammation.

## Discussion

We provide the first age-resolved human breast cancer landscape by defining transcriptomes, interactomes, and signaling pathway activity for TNBC and ER+ breast cancer at cell type-specific resolution. Our approach combined age-stratified differential gene expression analysis on bulk RNA-seq data, a new pipeline for identifying age-correlated gene sets from single-cell RNA-seq data (ASPEN), and cell interaction analysis to explore age-biased signaling in the TME. This integrated approach revealed molecular and cellular profiles that differentiate tumors from older and younger breast cancer patients in a subtype-dependent manner with important biological and clinical implications.

The approach uncovered novel insights not revealed by bulk transcriptional profiling. For example, while it is well accepted that systemic inflammation increases with age, the specific contribution of age-related inflammation in the TME of breast cancer remains unclear. We found distinct differences in inflammatory drivers between TNBC and ER+ breast cancer. In ER+, the strongest age-related inflammatory processes rested with myeloid cells, while in TNBC, inflammatory processes were primarily observed in CAFs. Moreover, in TNBC, SPP1-expressing macrophage communication was exclusive to younger patients, while in ER+ breast cancer, it was exclusive to older patients. Hence, inflammatory processes exhibited not only age-dependent but also subtype-dependent phenotypes.

The results underscore the risks of generalizing aging effects, reinforcing that aging varies not only across tissues and cancers^63^, but also within specific cancer subtypes. For example, increased EMT with age has been observed in pan-cancer bulk analyses^64^, and here, we noted EMT in specific cancer cells in the older TNBC cohort but not in ER+ breast cancer. One likely explanation is that EMT capacity and other age-related differences that we observed between subtypes are due to differences in tumor cell of origin and how they age. However, the differences in stromal and immune populations between subtypes suggest that their variations are influenced by aging in the context of subtype, not just aging alone.

The differences in immune responses between age and subtype are striking and warrant further investigation into the immune-modulatory role of various cells and their potential therapeutic implications with age. It is not clear why immunotherapies have limited efficacy in TNBC. The significant enrichment of immunosuppressive pathways in the older TNBC cohort suggests it may be easier to overcome immunosuppression in younger women than in older women. The groundwork we lay here - e.g., the strong immunomodulatory impact of CAFs - suggest novel ways to investigate intrinsic and acquired therapeutic resistance. In ER+ breast cancer, clinical data suggest that younger women benefit more than older women from addition of chemotherapy to endocrine therapy^65,66^, possibly due to higher immune responses in younger patients^17^, as we also observed here. This may indicate a subset of younger ER+ patients who could benefit from immunotherapy, despite its limited efficacy in this subtype^53^.

Tissue remodeling emerged as a key differentiator between age-related TNBC and ER+ breast cancer. This distinction holds potential for the design of subtype-specific therapies tailored to age-related profiles. For example, laminin-binding integrins, which govern cell morphology, polarity, differentiation, and migration, showed age-biased subunit variations in ER+ breast cancer, offering therapeutic targets. Additionally, age-related vascular remodeling, which influences immune infiltration and drug accessibility, represents another potential therapeutic avenue, guided by age-associated factors.

Areas of concordance between the two datasets strengthen the reliability of our results, while further validation in additional datasets and experimental models is warranted. Our study provides a robust foundation for such future efforts, offering a valuable resource for generating hypotheses, supporting orthogonal experimentation, and inspiring deeper investigations into age-associated changes in the TME that affect progression and drug response. The sample size limitation and lack of patient-specific outcomes in the single-cell RNA-seq cohort will be resolved as larger single-cell atlases become public in the future. Furthermore, since menopause is an important part of aging, it is reasonable to assume that some of our observations, particularly in ER+ breast cancers, are driven by the menopausal status of the donors. Future investigation into datasets that include well-annotated menopausal status might distinguish age-related differences driven by menopause from those driven by other aging processes. Importantly, our study provides a framework that can be applied to other datasets, not only in breast cancer, but any tumor type with single-cell RNA-seq data from young and older donors.

Our study demonstrates that the breast cancer TME differs profoundly with age in a subtype-specific manner. These findings establish a new framework and suggest that efforts to gain deeper insights into breast cancer pathology and design improved therapies should take age into consideration in a subtype-specific manner. Ultimately, integrating cell type-resolved methods to study the biology of TNBC and ER+ breast cancer across age groups is likely to result in tailored therapies that target specific tumor vulnerabilities and improve patient outcomes.

## Online methods

### Differentially expressed genes in younger and older breast cancer patients

Gene expression from donors from the METABRIC bulk RNA expression database with an age, three-gene disease subtype, and tumor stage were grouped into ER+ or TNBC and by age as <45 years and >65 years. For each of these 4 groups, donors were subsetted to include those whose tumors were Stage I - Stage III at diagnosis. Median age for the >65 TNBC group was 70.6 years (n= 63). The <45 TNBC group had a median age of 39.77 years (n = 50). Median age for the >65 ER+ group was 72.81 years (n= 386). The <45 ER+ group had a median age of 41.14 years (n = 86).

Log_2_-normalized METABRIC gene expression data and associated metadata were downloaded from cBioPortal. For each breast cancer subtype, genes (n = 24,174 total genes), were first subsetted to the top 675 genes that had the highest variance by ranking genes by standard deviation. The data were then transformed by exponentiating by base 2. The limma R package was used alongside voom normalization to identify differentially expressed genes between donors <45 years and >65 years in the TNBC and ER+ cohorts. We set the FDR significance threshold for both cohorts to 0.05. All 675 genes were then plotted in a volcano plot with log_2_ Fold Change on the x-axis, and −log_10_ FDR on the y-axis.

The total list of 675 genes was ranked by log_2_FC, starting with highest positive (most enriched in >65) and ending with lowest negative (most enriched in <45), and then GSEA was performed using the fgsea package on the C2, C5, and Hallmark pathway gene sets (MSigDB). Pathways with FDR < 0.05 for TNBC and ER+ were then visualized.

### ASPEN: Hallmark pathway correlation with age at cell type resolution using single-cell RNA sequencing data

We developed ASPEN (**A**ge-**S**pecific activation **P**rogram **EN**richment) to assess correlation between gene set (pathway) expression and age at cell type resolution. Single-cell RNA-seq counts matrices, barcodes, feature data, and metadata for 10 TNBC and 11 ER+ primary breast tumors were downloaded from GEO (GSE176078), and additional cohort metadata (including age annotations) were downloaded from the accompanying manuscript’s supplementary data^24^. Following standard pipelines in Seurat v4, a Seurat object was made for each of the 21 samples and the 10 TNBC donors’ objects and 11 ER+ donors’ objects were merged into a single TNBC object and a single ER+ object. From there, the data were log-normalized and parallel adaptations of traditional gene set enrichment analyses (GSEA) were performed to assess correlation between donor age and Hallmark Pathway enrichment per cell type. The middle-granularity cell type annotations provided by the authors of the dataset were used (29 total cell types, celltype_minor).

For the first arm of ASPEN, average gene expression per cell type was correlated to donor age and GSEA was performed on the genes in the Seurat object ranked by correlation coefficient from most correlated to most anti-correlated. The TNBC or ER+ merged objects were re-subsetted by donor. Within each of the 10 (TNBC) or 11 (ER+) objects, average gene expression for each of the 29 cell types was calculated on a per gene basis. Some donors had a cell count of 0 for specific cell types; these donors were excluded from the next analysis steps. If more than half of the donors had a given cell type, the average expression values for each gene for that cell type were then correlated to donor age (a total of 24 unique cell types for TNBC and 25 unique cell types for ER+). Each gene per cell type was then ranked from highest correlation coefficient (most correlated) to lowest (most anti-correlated). To avoid erroneous results due to arbitrary gene ranking, the genes with a correlation coefficient of 0 were omitted, and the remaining ranked genes were used to perform GSEA for the Hallmark pathways. GSEA was performed using the fgsea and gage R packages; this portion of the script was adapted from a publicly available GSEA script developed by Dr. Brian Gudenas (https://bioinformaticsbreakdown.com/how-to-gsea/). Gene set .gmt files were accessed from the GSEA website. For a given cell type/pathway combination to be considered a statistically significant enrichment, an adjusted p-value <0.05 in both package analyses was required. The statistically significant cell type/pathway combinations were then visualized as bubble color and depth in a bubble plot.

For the second arm of ASPEN analysis, enrichment for each cell within the dataset was first performed and then the numeric metric of enrichment was correlated to donor age. For this analysis, the TNBC or ER+ merged objects were re-subsetted by donor and the *AddModuleScore* command within Seurat v4 was used to assign a signature score to each cell for gene expression concordance with the 50 Hallmark pathways. These gene sets were accessed in R using the msigdbr R package. Once every cell per donor had a signature score, the average signature score per cell type was calculated and the resulting average signature score per cell type per donor was correlated to donor age. The magnitude of these correlation coefficients was then visualized as bubble size in a bubble plot.

### Cell-cell interaction analysis

For the analysis of cell-cell interactions, we used CellChat (version 2.1.2) ^29^.The single-cell human breast cancer atlas data was divided into four groups according to subtype and patient age at diagnosis (Supplementary Table 5). We created four CellChat objects and associated datasets, one for ≤55 TNBC donors, one for >55 TNBC donors, one for ≤55 ER+ donors, and one for >55 ER+ donors, from the single-cell RNA-seq data. To consider the proportion of cells in each group when calculating the cell-cell interaction probability, the *population.size* argument in the *computeCommunProb* function was set to *TRUE*. In addition, we used *liftCellChat* when the cell type populations were different between young and old CellChat objects by subtype, as was the case in ER+ ≤55 where there were no Naive B cells. Then, for each subtype, we merged the CellChat objects to run a comparison analysis between young and aged groups. The analysis allowed us to determine the number and strength of interactions between cell types in the different cohorts and to visualize them using different tools, such as circle plots (*netVisual_circle* and *netVisual_diffInteraction*), scatter plots (*netAnalysis_signalingRole_scatter*), bar charts (*rankNet*), or bubble blots (*netVisual_bubble*). Circle plots show overall interaction probabilities between cell types of interest, either per group or differentially between groups. The scatter plots show changes in both incoming and outgoing interaction strengths for each cell type individually, where interaction strength represents the sum of incoming and outgoing probabilities for all cell-cell interactions. The bar charts describe statistically significant, pathway-specific differences between a given source and target cell group and determine statistical significance between donors ≤55 and >55. The bubble plot calculates the communication probability of each ligand-receptor interaction between source and target cells in each age cohort for a given pathway or pathways. Fold change differences above 1.2 or below −1.2 were of interest.

### Regression-based criteria for curating cells and signaling nodes

Cell types of focus were chosen based on source and/or target signal strengths greater than the sum of the average source/target interaction strengths of the younger and older cohorts (>0.037 for TNBC and >0.023 for ER+ breast cancer, Supplementary Table 6). The criteria yielded 7 cell types for TNBC: iCAF, myCAF, basal cancer cells, macrophages, monocytes, CD4+, and CD8+ T cells. For ER+ breast cancer, these criteria revealed 8 cell types: iCAF, myCAF, cancer luminal A, macrophages, ACKR1+ endothelial cells, differentiated PVLs, CD4+ T cells, and CD8+ T cells. We excluded three cell types despite meeting the criteria: 1) TNBC cancer HER2 cells because only one patient sample contained appreciable numbers of cancer HER2 cells (Supplementary Fig. 2a); 2) TNBC cycling cancer epithelial cells because we did not know their intrinsic molecular subtype; and 3) ER+ breast cancer luminal B cells because the luminal A cells had a 6.7-fold higher interactome enrichment with age (Supplementary Table 6).

We then applied the *rankNet* function to these 7 or 8 cell types as both source cells and target cells (49 total interactions for TNBC and 64 total interactions for ER+) to identify signaling pathways through which these cell types were interacting. The *rankNet* function utilizes inferred communication probabilities between a given source cell and a target cell for 229 different signaling pathway categories (totaling 3,234 ligand-receptor pairs) and yields the scaled communication weight for every inferred signaling pathway. The probabilities of specific ligandreceptor interactions (“signaling nodes”) for each signaling pathway category identified in the *rankNet* analysis were extracted for further investigation.

To select the most relevant pathways for downstream analysis, we then performed a univariate logistic regression model with the glm function of the stats R package. The logistic regression for each pathway assessed how well it was associated with the older cohort, with each source-target combination for both the young and older cohorts serving as a data point. To integrate the data and identify key signaling pathways, we nominated signaling pathways that were ubiquitous across the TME as they were age-differential (p < 0.05) and appeared at least 15 times across the total signaling network by rankNet analysis. We first averaged the probabilities of interaction for each ligand-receptor pair in each pathway, source cell, and target cell interaction for the >55 or <55 age groups. These average communication probabilities were used as input to the glm function, where each pathway was the test variable and age group was the response variable. We then selected pathways that had a p-value <0.05 and appeared at least 15 times in the 49 or 64 interactions analyzed using *rankNet*. We used the *netVisual_bubble* function a second time to compare the up-regulated and down-regulated ligand-receptor pairs between age groups in both molecular subtypes, setting a p-value threshold <0.01 to highlight the ligand-receptor pair interactions with the highest confidence. For these, we identified signaling interactions that were either exclusive to one age group or exhibited fold change differences above 1.2 or below −1.2 between age groups.

In some cases where indicated, we nominated additional select signaling nodes by manual curation of significant rankNet interaction pathways that are: 1. known to modulate cell states and phenotypes that we observed in the METABRIC dataset or ASPEN analyses, 2. evidence-based age-related factors, or 3. highly age-biased but restricted to specific cell:cell interactions (i.e., unable to meet our selection criteria of >15 interactions).

## Data Availability

METABRIC gene expression data was accessed through cBioPortal. The single-cell RNA sequencing data used in this study is publicly available and was accessed through GEO Accession number GSE176078.
